# Virulence of *Candida albicans* isolated from HIV infected and non infected individuals

**DOI:** 10.1186/s40064-015-1215-0

**Published:** 2015-08-11

**Authors:** Tri Wibawa, Praseno, Abu Tholib Aman

**Affiliations:** Department of Microbiology, Faculty of Medicine, Universitas Gadjah Mada, Jl. Farmako, Sekip Utara, Depok, Sleman, Yogyakarta, 55281 Indonesia

**Keywords:** *C. albicans*, Virulence factors, HIV infection

## Abstract

*Candida* sp contributes 33.1 % of fungal infections among HIV patients. Among the species of the genus Candida, *Candida albicans* is the most frequently isolated from HIV patients. This study aimed to analyze putative virulence factors of *C. albicans* isolated from oral cavities of HIV infected patients and healthy individuals. Twenty isolates from HIV infected patients and fourteen from healthy individuals were analyzed for phenotypic switching, cell growth rate, hyphae formation, hemolytic activity and biofilm formation characteristics. The frequency of phenotypic switching was low in both groups. The cell growth rate of *C. albicans* from HIV infected patients were significantly higher than those from healthy individuals (p < 0.001). After 48 h incubation, the concentration of *C. albicans* isolated from HIV infected patients was 8.6 × 10^6^ cells/ml while the concentration of *C. albicans* isolated from healthy individuals was 7.8 × 10^6^ cells/ml. After 72 h incubation, the concentration of *C. albicans* isolated from HIV infected patients was 9.5 × 10^6^ cells/ml while the concentration of *C. albicans* isolated from healthy individuals was 8.2 × 10^6^ cells/ml. In contrast, the hemolytic activity of *C. albicans* isolated from healthy individuals were significantly higher compared to those from HIV infected patients (p < 0.001) at both aerobic (6 vs. 3.5 mm) and anaerobic (3.8 vs. 1.3 mm) conditions. The percentages of hyphae forming cells were higher in *C. albicans* collected from HIV infected patients (27.5 %) compared to the healthy individual group (14.7 %). However, this trend was not statistically significant (p = 0.1). *Candida albicans* isolated from HIV infected patients have similar ability to develop biofilms compared to those from healthy individuals. (OR = 4.2; 95 % CI 0.724–26.559). The virulence factors of *C. albicans* isolated from HIV infected patients were not significantly different from those of healthy individuals. The results add new insights into the contribution of virulence factors in the pathogenesis of *C. albicans* infection in HIV infected patients.

## Background

HIV/AIDS is now a major global health problem. In 2012, there were 1.6 million AIDS related deaths and at least 32 million adults and children were living with HIV globally. The situation is still far from the global vision of zero new HIV infections, zero discrimination and zero AIDS-related deaths (UNAIDS 2013). Fungal infections remain a clinical challenge in HIV patients with severe immune suppression conditions. The *Candida* species contributes up to 33.1 % of fungal infections among HIV diagnosed individuals. CD4+ cell counts <200 cells/μl and other underlying conditions in HIV patients were found susceptible to invasive candidiasis (Marukutira et al. 2014) with *C. albicans* being the most frequently isolated (Fidel 2011; Anwar et al. 2012; Okonkwo et al. 2012).

The *Candida* species are endogenous microbes in several organ systems, including the mouth and the intestines. While these fungi are commonly considered to be non pathogenic, they are responsible for opportunistic infections. Candida may alter from the commensally harmless to a pathogenic state. This may occur following alteration of the oral cavity environment thus favoring its growth (Williams and Lewis 2011). There are several clinical presentations related with *C. albicans* infection in the oral cavity of HIV patients; i.e. xerostomia, oral hairy leukoplakia, necrotizing ulcerative periodontitis, Kaposi’s sarcoma, human papilloma virus-associated warts, and other ulcers (Reznik 2006).

Several putative virulence factors of *C. albicans* have been reported such as phenotypic switching, hyphae formation, biofilm formation, esterase production, phospholipase expression, proteinase production, hemolytic activity, adhesin activity and invasin (Mayer et al. 2013). However, only few of these putative virulence factors were studied in HIV infected patients. Interestingly, all *C. albicans* isolates from HIV infected patients were shown to produce intermediate to high levels of proteinase and phospholipase (Menezes et al. 2013). Biofilm formation was reported to be similar between *C. albicans* isolates from HIV infected patients and healthy individuals (Jin et al. 2003) and the phenotypic switching of *C. albicans* was considered high in HIV infected patients (Vargas et al. 2000). It is thus important to study the pathogenesis of *C. albicans* in HIV infected patients. The immune response which is compromised in HIV infected patients or virulence factors of *C. albicans* which is working as underlying mechanism, are interesting subjects to be elucidated.

This study aimed to analyze some putative virulence factors of *C. albicans* isolated from the oral cavity of HIV infected patients and healthy individuals. Our results showed that some of the virulence factors are significantly different among those two series of *C. albicans*. This result may add new insights to the contribution of virulence factors in the pathogenesis of *C. albicans* infection in HIV infected patients.

## Methods

### *C. albicans* isolates

This study made use of archived *C. albicans* isolates. A total of thirty-four (34) *C. albicans* isolates were obtained. Twenty (20) isolates were from HIV infected patients and fourteen (14) isolates came from healthy individuals. *Candida albicans* was cultured from rinsed oral cavity fluids of HIV infected patients and healthy individuals. *Candida albicans* identification was performed using chromogenic CHROMagar™ Candida. The *C. albicans* isolates were subsequently cryo-preserved at −80 °C until further analysis.

A unique code was assigned for every isolate to protect the identities of the patients and the healthy volunteers. The protocol received approval from the Medical and Health Research Ethics Committee of the Faculty of Medicine, Universitas Gadjah Mada, Yogyakarta, Indonesia.

### Phenotyphic switching induction

Frozen *C. albicans* isolates were thawed and inoculated on yeast extract peptone dextrose (YPD) solid medium and incubated for 3 days at 25 °C. A colony from each isolate was taken from the YPD medium, again inoculated on YPD, and incubated for 3 days at 25 °C. One to five single colonies were diluted with 200 μl sterile water, and adjusted to McFarland 0.5 standard to get 1 × 10^6^ CFU/ml concentrations. The yeast suspension was inoculated on YPD medium supplemented with 50 μg/ml phloxin B, and incubated for 3 days at 25 °C. The opaque cells produced red colonies and the white cells were regular white colonies. Cell morphology was confirmed using a light microscope after lacto phenol cotton blue staining. The opaque cells appeared dark and oval while the white cells appeared bright and spherical. To define the frequency of phenotypic switching, each isolate was cultured on YPD medium supplemented with phloxin B. This was done for at least 4,000 colonies (Hnisz et al. 2011).

### Cell growth rate

*Candida albicans* was grown on Saboroud’s Dextrose Agar (SDA) supplemented with 5 % chloramphenicol. Several colonies of *C*. *albicans* were inoculated into casein-yeast-glucose (CYG) broth. The cell concentration was measured using a spectrophotometer at 540 nm. Initial concentrations of all *C. albicans* isolates were calibrated into equal optical density (OD), and considered as zero minute concentrations. The yeast suspensions were then incubated at room temperature. Cell concentrations were measured serially at 0, 12, 24, 48, and 72 h after inoculation. The growth curves of yeast were obtained from duplicate measurements using a spectrophotometer. The conversion of OD to cell concentration was performed according to the formula published by Rodrigues et al. (1993)

$${\text{Cell concentration }} = \, \left\{ {64.3 \, + \, \left( {8.206 \, \times \,} \mathrm{OD} \right)} \right\} \, \times \, 10^{3} {\text{cells}}/{\text{ml}} .$$

### Hyphae formation

*Candida albicans* was inoculated into fetal bovine serum (FBS) and incubated for 90 min at 37 °C. After incubation, the cells were counted using a hemocytometer cell counter (Rodrigues et al. 1993). The total number of cells and hyphae forming cells were determined and the percentages of hyphae forming cells were calculated. Duplicate independent cell counting was done.

### Hemolytic activity

Yeast suspension was prepared from several colonies of *C. albicans* and was adjusted to McFarland 0.5. Ten (10) μl of yeast suspension was dropped on SDA supplemented with 5 % chloramphenicol, 7 % sheep blood and 3 % glucose, and incubated at 37 °C for 48 h in both aerobic and anaerobic condition as described by Inci et al. (2012). After incubation, the transparent zone around the colonies was considered the hemolytic zone. The diameter of both hemolytic zones and the colonies were then measured. The hemolytic zone diameter was subtracted from the diameter of the colonies and was documented as the hemolytic activity level in this study. Triplicate independent experiments were done.

### Biofilm formation

*Candida albicans* was inoculated into YPD broth and incubated for 24 h at 37 °C. Then, the yeast suspension was washed and centrifuged three times at 3,000 rpm for 10 min. The pellet was diluted with RPMI 1640 and the cell concentration was adjusted to 10^6^ CFU/ml. The suspension was transferred into 96 wells polystyrene microplates with as much as 200 μl for each well. This was then incubated for 48 h at room temperature. Each *C. albicans* isolate was cultured into eight wells to have eight replication results. The wells were washed twice with 10 mM PBS. After washing, 50 μl 1 % crystal violet was added into the wells and kept at room temperature for 30 min. Water was used to rinse the wells for three times. Finally, 200 μl acidic isopropanol was added into the wells and kept at room temperature for 10 min. The ODs were measured using the ELISA reader at 595 nm (Thermo Scientific). The cut-off point of the biofilm forming *C. albicans* was calculated using the formula: 3 × StDev + OD of control. The control is considered as the well that was added with broth medium without *C. albicans*. The *C. albicans* with OD less than the cut-off was considered as non adhered (non biofilm forming); less than 2 times of the cut-off was considered as weakly adhered; 2–4 times of the cut-off was considered as moderately adhered; and more than 4 times the cut-off was considered as strongly adhered (Stepanovic et al. 2004). The data of each isolate were obtained from 8 replicate wells of duplicate independent experiments. Only 12 out of 14 *C.albicans* isolated from healthy individuals were analyzed for biofilm formation assay.

### Statistical analysis

The Wilcoxon sum-of-ranks test was used to analyze the difference between the two groups of *C. albicans*.

## Results

**Fig. 1:**
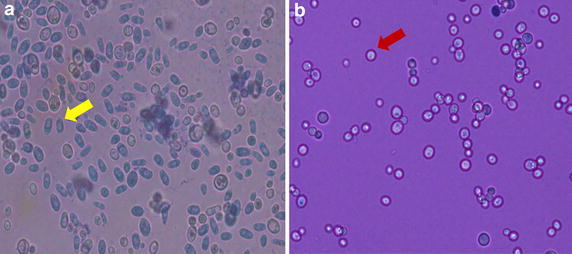
Microscopic examination of *C. albicans* after lacto phenol cotton blue staining. **a** Cd32 underwent white-opaque switching; Opaque cells (*arrow*) appear oval, dark, and polymorphic. **b** White cells (*arrow*) appear spherical and bright. The observations were done in ×400 magnification.

**Fig. 2:**
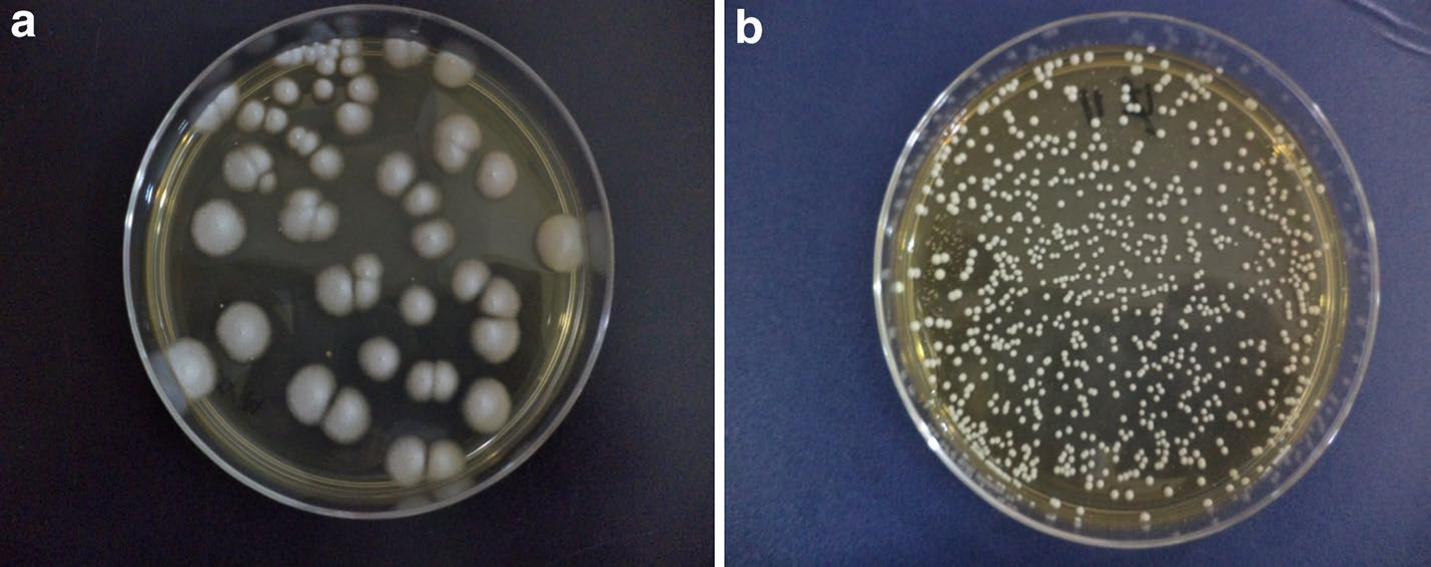
*C. albicans* colonies on yeast extract peptone dextrose (YPD) agar. **a** Colony morphology of Ck74, isolated from a healthy individual, has morphological switching. The colonies appear white, velvety and bigger. **b** Representative colonies of other *C. albicans* isolates appear glistening, domed shaped, and smooth.

Twenty (20) *C. albicans* isolates from HIV infected patients and 14 *C. albicans* isolates from healthy individuals were studied. Among 34 *C. albicans* isolates, only one isolate showed phenotypic switching (alteration from white to opaque cells) after induction on YPD medium. This particular isolate (Cd32) was from an HIV infected patient. The yeast cells looked darker and oval compared to the usual *C. albicans* that is bright and spherical in shape after lacto phenol cotton blue staining (Fig. 1). The frequency of the phenotypic switching for the Cd32 isolate was 5/4,000 colonies. The variation of the *C. albicans* colony morphology was observed in Ck74 which was isolated from a healthy individual. This isolate showed colonies which were not glistening, domed shaped, and smooth as usually seen in a normal *C. albicans* colony morphology. It showed white, velvety and bigger colonies (Fig. 2).

**Fig. 3:**
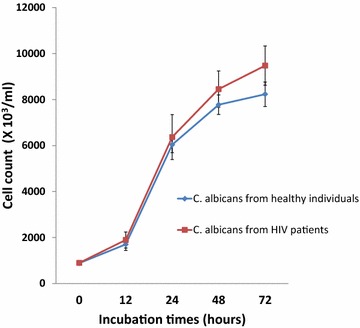
Growth rate of *C. albicans*. Cells concentration was measured using a spectrophotometer at 540 nm. Data were obtained from duplicate measurements for each isolate. After 48 h incubation, the cell concentration of the *C. albicans* collected from HIV infected patients were significantly higher than those collected from healthy individuals (p < 0.001).

The growth rate of the two series of *C. albicans* was studied. The cell concentrations were measured using a spectrophotometer at 540 nm, and the OD was than deducted from the cell concentration. These observations were performed serially on the time incubation-based measurements. After 48 h incubation, the growth curves were significantly different between the two series (p < 0.001). The *C. albicans* isolated from HIV infected patients grew faster than those isolated from healthy individuals, as shown by the higher cell concentration after 48 h incubation. After 48 h incubation, the concentration of *C. albicans* isolated from HIV infected patients was 8.6 × 10^6^ cells/ml while the concentration of *C. albicans* isolated from healthy individuals was 7.8 × 10^6^ cells/ml. After 72 h incubation, the concentration of *C. albicans* isolated from HIV infected patients was 9.5 × 10^6^ cells/ml while the concentration of *C. albicans* isolated from healthy individuals was 8.2 × 10^6^ cells/ml (Fig. 3).

**Fig. 4:**
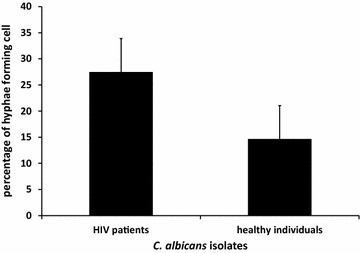
The percentages of hyphae forming cells of *C. albicans* isolated from HIV infected patients were not different from those isolated from healthy individuals (p = 0.1). The data were obtained from duplicate independent cell counting.

The ability of the two series of *C*. *albicans* to form hyphae was studied by measuring the proportion of cells that develop hyphae after incubation in the fetal bovine serum at 37 °C for 90 min. The percentages of hyphae forming cells were higher in *C. albicans* collected from HIV infected patients (27.5 %) compared to the healthy individual group (14.7 %). However, this trend was not statistically significant (p = 0.1) (Fig. 4).

**Fig. 5:**
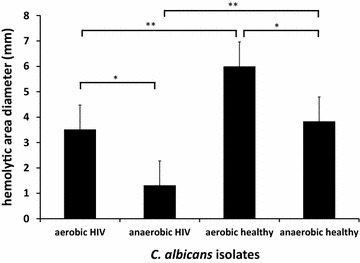
Hemolytic activity of *C. albicans* isolated from HIV infected patients were significantly lower than isolates obtained from healthy individuals in both aerobic and anaerobic conditions (**) (p < 0.001). The two series of *C. albicans* showed significantly higher hemolytic activities in aerobic conditions (*) (p < 0.05). The data were obtained from triplicate independent experiments.

The hemolytic activity of *C*. *albicans* from healthy individuals was significantly higher compared to the series from HIV infected patients (p < 0.001) at both aerobic (6 vs. 3.5 mm) and anaerobic (3.8 vs. 1.3 mm) conditions. It was noted also that the hemolytic activity of all *C. albicans* isolates was higher in aerobic (4.5 mm) than anaerobic (2.3 mm) conditions in both series of *C. albicans* (p < 0.05) (Fig. 5).

The cut-off points of both biofilm forming and non biofilm forming abilities were defined according to the formula: (3StDev + mean of the control). The cut off was OD = 0.346. The distribution of the biofilm forming characteristic of the *C. albicans* isolates is shown in Table 1. The isolates with OD similar or less than the cut-off were considered non adhered or non biofilm forming. The isolates with OD exceeding the cut-off were considered as able to adhere (weak, moderate and strong) or biofilm forming. The data were introduced into a 2 × 2 contingency table and the Odds Ratios were subsequently computed. The ability of the two series of *C. albicans* to develop a biofilm seemed not significantly different. *C. albicans* isolated from HIV infected patients have the same frequency of developing biofilms compared to those isolated from healthy individuals (OR = 4.2; 95 % CI 0.724–26.559) (Table 1).

**Table 1:** The biofilm forming characteristics of *C. albicans* according to its origin

Origin of *C. albicans*	Non biofilm forming	Biofilm forming			Total number of isolates
		Weakly adhered	Moderately adhered	Strongly adhered	
HIV infected	5	7	6	2	20
Healthy individual	7	1	3	1	12
Total	12	8	9	3	32

## Discussion

Our data showed that phenotypic switching, white-opaque cells and the colony morphology change are not frequently found among *C. albicans* isolated from HIV infected patients and also from healthy individuals. Previous reports showed that there were high frequencies of phenotypic switching among *C. albicans* isolated from HIV infected patients (Vargas et al. 2000). The discrepancy may have resulted from the diversity of *C. albicans* circulating in different geographical areas and also from different study methods. It is well known that phenotypic switching in *C. albicans* is a complex mechanism influenced by environmental conditions such as CO2 concentration, temperature, and nutrient availability (Huang et al. 2009; Alby and Bennett 2009). Our data suggests that the frequency of phenotypic switching was similar between *C. albicans* isolated from both the HIV infected and healthy individual groups.

The high prevalence of azole resistant *C. albicans* isolated from an HIV infected patient has been documented (Dos Santos Abrantes et al. 2014). This phenomenon is aggravated by the presence of a biofilm which is naturally resistant to antifungal agents. The *C. albicans* cell growth rate has positive correlation with the ability to adhere and colonize at the surface of epithelial cells of the oral cavities of patients (King et al. 1980). Furthermore, cell density has a role in the phenotypic resistance of a biofilm to antifungals (Perumal et al. 2007). Figure 3 showed that the *C*. *albicans* collected from HIV infected patients have significantly higher cell growth rate than those isolated from healthy individuals. This result suggests that *C. albicans* isolated from HIV infected patients are more virulent compared to isolates taken from healthy individuals.

It should be noted that Cd32, the isolate that showed white-opaque switching, had the highest growth rate among all other *C. albicans* isolates. Meanwhile, the growth rate of Ck74 which showed colony morphology switching, is similar with other isolates. This is not in accordance to a previously reported study where it was reported that in some mutant *C. albicans*, the cellular growth rate has a negative correlation with white-opaque switching (Alby and Bennett 2009). However, because of the low frequency of white-opaque switching in our experiments, the authors could not further confirm the relationship between the two variables.

*Candida albicans* is able to form pseudohyphae and true hyphae. Hyphae formation is considered an important virulence factor that facilitates yeast to become pathogenic in humans (Lo et al. 1997; Mayer et al. 2013). The hyphae of *C. albicans* may have the same properties of other fungal hyphae such as, the ability to exert mechanical force, facilitate penetration of the epithelial surface, and damage endothelial cells. These are the characteristics of hyphae as a virulence factor (Kumamoto and Vinces 2005). Our data showed that the hyphae formation of *C. albicans* collected from HIV infected patients were not different from those of healthy individuals. It is suggested that the *C. albicans* from HIV infected patients are similar with healthy individuals in terms of virulence.

The hemolytic activity of *C. albicans* is considered a virulence factor that influences the pathogenicity of the yeast. Some metals were found to be essential for various cellular protein and enzyme functions such as iron, zinc, manganese and copper (Mayer et al. 2013). *Candida albicans* expresses a hemolytic factor which allows it to acquire iron from host erythrocytes. *Candida albicans* may acquire this iron by producing factors which can release hemoglobin by lysing erythrocytes (Manns et al. 1994). Our results showed that all *C. albicans* isolates have hemolytic activities as previously reported by Mane et al. (2011). The hemolytic activity of *C. albicans* isolated from HIV infected patients was lower compared to the isolates from healthy individuals. It may be suggested that *C. albicans* from HIV infected patients are less virulent.

*Candida albicans* remains the fungal species most commonly associated with biofilm formation (Ramage et al. 2005). Our results showed that only 42 % of *C. albicans* isolated from healthy individuals were able to form a biofilm (consist: weakly–moderate–strongly adhered). In contrast, 75 % of *C. albicans* from HIV infected patients were biofilm forming isolates (Table 1). However, 2-way contingency table analysis did not confirm that *C. albicans* isolated from HIV infected patients were more virulent (OR = 4.2; 95 % CI 0.724–26.559). The authors prefer to use the cut-off value of OD to describe the biofilm formation ability among the two groups of *C. albicans*. Using this approach, the authors can evaluate which isolates actually adhered to the polystyrene surface. This result is in agreement with a previously published report stating that, biofilm formation of *C. albicans* was not considered different between HIV infected patients and healthy individuals (Jin et al. 2003).

Biofilm formation has an important role in the development of clinically resistant *C. albicans* against antifungal treatment (Kuhn et al. 2002; Mukherjee et al. 2003). Resistance to antifungals may result in higher morbidity and mortality since infections with antifungal resistant strains are very difficult to manage in the clinics. Biofilm formation is influenced by hyphae formation, one of the important phases of biofilm development. Two *C. albicans* mutants showed that the hypha-negative mutant produced only the basal layer, and the yeast-negative mutant produced only the outer layer which was more easily detached from the catheter. This finding suggests that dimorphism might be necessary for biofilm architecture and structure (Kojic and Darouiche 2004). Our data showed that *C. albicans* isolated from HIV infected patients have similar ability to develop biofilms compared to those from healthy individuals. This result paralled with our data showing similar hyphae formation but not with significantly higher cell growth of *C. albicans* isolated from HIV infected patients.

In conclusion, the role of virulence factors of *C. albicans* in the opportunistic infection pathogenesis collectively with the immunocompromised condition of HIV infected patients is not yet clearly elucidated. The virulence factors of *C. albicans* may not be working in the same manner to facilitate infection in HIV infected patients and also in healthy individuals. It is also recognized that there are various factors regulating the pathogenecity of *C. albicans*.
